# System dynamics modeling of childhood obesity

**DOI:** 10.1186/1471-2105-13-S12-A13

**Published:** 2012-07-31

**Authors:** Behrouz Madahian, Robert C Klesges, Lisa Klesges, Ramin Homayouni

**Affiliations:** 1 Bioinformatics Program, University of Memphis, Memphis, TN 38152, USA; 2 Department of Preventive Medicine, University of Tennessee Health Science Center, Memphis, TN 38163, USA; 3 Department of Epidemiology and Social and Behavioral Sciences, University of Memphis, Memphis, TN 38152, USA; 4 Department of Biology, University of Memphis, Memphis, TN 38152, USA

## Background

Effective strategies for prevention of obesity have been elusive since the recognition of obesity as a major public health issue [1]. Obesity is a result of chronic, quantitative imbalance between energy intake and energy expenditure, influenced by a combination of genetic, environmental, psychological and social factors. Therefore, a systems perspective is needed to examine effective obesity prevention strategies [2]. In this study, a system dynamics model was developed using the data from the Girls health Enrichment Multi-site Studies (GEMS). GEMS tested the efficacy of a 2-year family-based intervention to reduce excessive increase in body mass index in 8-10 year old African-American girls.

## Methods

**Figure 1:**
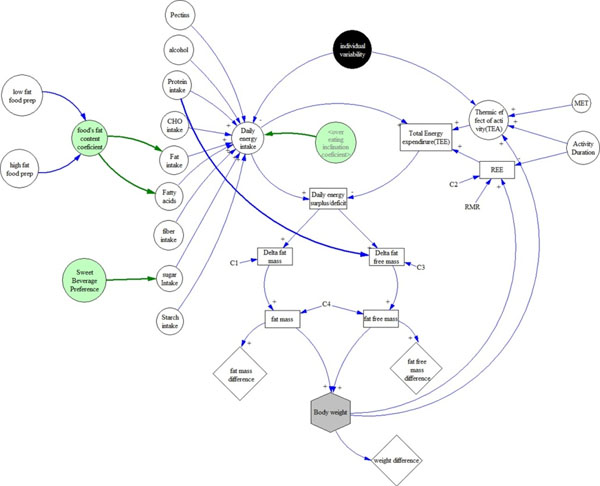
The complete system dynamic body weight model included sugar and fat intake variables (open circles) as well as environmental and behavioral subsystems (green circles).

System dynamics models were built using Vensim software (Ventana Systems, Inc) [3]. First, a core model was built which contained energy intake, energy expenditure and energy surplus subsystems contributing to the total body weight [4,5]. Then, an optimum model was built by systematically adding variables (from among 313 collected in GEMS) to fit the observed data by regression analysis for 50 randomly selected individuals from the cohort. The final model included nutrition, physical activity, and several environmental and behavioral factors (Figure 1).

## Results and discussion

**Figure 2:**
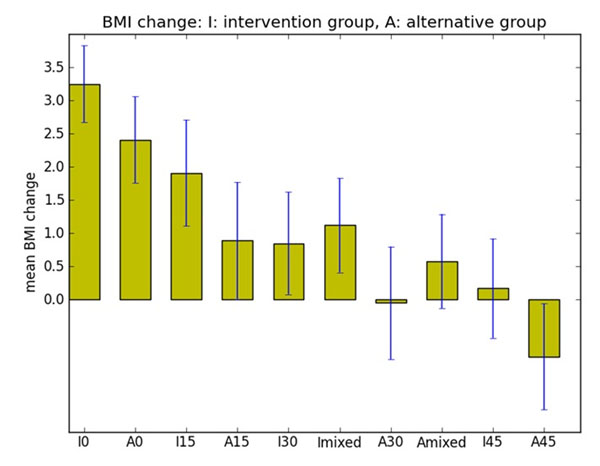
Comparison of various intervention schemes on simulated BMI change. The GEMS cohort consists of an intervention group (I) and an alternative group (A). Different durations (0, 15, 30 and 45 min) of exercise were simulated for both groups as denoted on the x-axis. In addition, a ‘mixed’ intervention was simulated which included 10 min of exercise with a 100 cal *reduction* in food intake.

We evaluated the performance of a large number of models containing different food intake, energy expenditure and environmental variables collected in GEMS. The BMI simulated by our final complete model showed a correlation coefficient of 0.83 with the observed data, whereas the initial core model showed a correlation of 0.55. The model accuracy was within 10% for 84% of the individuals in the study. For a few individuals, the simulated BMI and observed BMI values were greatly (> 35%) different. It is possible that the data collected from these individuals were inaccurate or, alternatively, it is possible that these individuals had other confounding factors (e.g. genetic variability) that were not captured in the study. Consistent with previous observations, we found that the two intervention strategies in the GEMS study did not affect the BMI increases observed in the cohort [1]. Interestingly however, using our model, we were able to compare various intervention strategies and found that 10 min exercise plus a reduction of intake by 100 calories produced the same effect on BMI change as 30 min of exercise per day (Figure 2). Our work suggests that system dynamics modeling may be useful for testing potential intervention strategies in complex disorders such as obesity.
